# Contributions of MET activation to BCR-ABL1 tyrosine kinase inhibitor resistance in chronic myeloid leukemia cells

**DOI:** 10.18632/oncotarget.16314

**Published:** 2017-03-17

**Authors:** Masanobu Tsubaki, Tomoya Takeda, Toshiki Kino, Kazuko Sakai, Tatsuki Itoh, Motohiro Imano, Takashi Nakayama, Kazuto Nishio, Takao Satou, Shozo Nishida

**Affiliations:** ^1^ Division of Pharmacotherapy, Kindai University School of Pharmacy, Kowakae, Higashi-Osaka, Japan; ^2^ Department of Genome Biology, Kindai University School of Medicine, Osakasayama, Osaka, Japan; ^3^ Department of Food Science and Nutrition, Kindai University School of Agriculture, Nara, Nara, Japan; ^4^ Department of Surgery, Kindai University School of Medicine, Osakasayama, Osaka, Japan; ^5^ Division of Chemotherapy, Kindai University School of Pharmacy, Kowakae, Higashi-Osaka, Japan; ^6^ Department of Pathology, Kindai University School of Medicine, Osakasayama, Osaka, Japan

**Keywords:** chronic myeloid leukemia, imatinib resistance, MET, ERK1/2, JNK

## Abstract

Resistance to the breakpoint cluster region-abelson 1 (BCR-ABL1) tyrosine kinase inhibitor (TKI) imatinib poses a major problem when treating chronic myeloid leukemia (CML). Imatinib resistance often results from a secondary mutation in BCR-ABL1. However, in the absence of a mutation in BCR-ABL1, the basis of BCR-ABL1-independent resistance must be elucidated. To gain insight into the mechanisms of BCR-ABL1-independent imatinib resistance, we performed an array-based comparative genomic hybridization. We identified various resistance-related genes, and focused on MET. Treatment with a MET inhibitor resensitized K562/IR cells to BCR-ABL1 TKIs. Combined treatment of K562/IR cells with imatinib and a MET inhibitor suppressed extracellular signal-regulated kinase 1/2 (ERK1/2) and c-Jun N-terminal kinase (JNK) activation, but did not affect AKT activation. Our findings implicate the MET/ERK and MET/JNK pathways in conferring resistance to imatinib, providing new insights into the mechanisms of BCR-ABL1 TKI resistance in CML.

## INTRODUCTION

Leukemia is a cancer of the bone marrow or blood characterized by an excessive increase in immature white blood cells. Chronic myeloid leukemia (CML) now accounts for 10-20% of all adult leukemias. The cause of CML is the Philadelphia chromosome, which results from a reciprocal translocation between the long arms of chromosomes 9 and 22, creating the chimeric breakpoint cluster region-abelson 1(BCR-ABL1) oncogene [1]. The BCR-ABL1 oncogene is a fusion protein that results from transposition of a segment of the c-ABL1 gene from chromosome 9q34 onto the BCR gene on chromosome 22q11. It encodes a cytoplasmic protein tyrosine kinase with elevated and dysregulated enzymatic activity that plays a vital role in the pathogenesis and progression of CML [2]. This fusion protein is found in approximately 95% of patients with CML and 30% of adult patients with acute lymphoblastic leukemia (ALL) [3]. Several mechanisms are involved in the malignant transformation orchestrated by the BCR-ABL1 oncoprotein. First, BCR-ABL1 activates a number of cell cycle control enzymes and proteins, accelerating cell division. In addition, BCR-ABL1 constitutively activates mitogenic signaling pathways, such as the Janus kinase/signal transduction and transcription (JAK/STAT) pathway, phosphatidylinositide-3 kinase (PI3K) pathway, RAS/mitogen-activated protein kinase (MAPK) pathways, and the MYC pathway [4].

The first-line treatment for CML is imatinib mesylate, which binds to the ABL1 kinase domain and inhibits phosphorylation of substrates. Although imatinib markedly improves patient survival when used to treat early-stage disease, the drug is not curative. Resistance to imatinib can develop, especially in advanced stages, leading to disease relapse and progression [5]. Resistance to imatinib arises from multiple mechanisms that can be classified broadly as either BCR-ABL1–dependent or BCR-ABL1–independent. BCR-ABL1–dependent resistance most commonly results from point mutations arising in the ABL1 kinase domain, which interfere with imatinib binding and subsequent kinase inhibition [6]. However, in 50% or more of imatinib-resistant CML patients, there is no mutation in BCR-ABL1, which the overexpression of BCR-ABL1 protein, SRC-family proteins, and ATP-binding cassette transporters, such as P-glycoprotein, is implicated in BCR-ABL1-independent imatinib resistance [7–14]. Additionally, activation of the PI3K/Akt pathway induces imatinib resistance [15], but the specific mechanisms that mediate BCR-ABL1-independent imatinib resistance are not well understood.

The present study investigates the mechanism(s) of imatinib resistance, and aims to determine whether alternative small molecule inhibitors can be used to overcome the limitations associated with imatinib in treating CML.

## RESULTS

### Sensitivity of imatinib-resistant cells to ABL1 tyrosine kinase inhibitors

To investigate imatinib resistance mechanisms, we established the imatinib-resistant K562/IR subline. Over a period of 6 months, K562 cells in culture were continuously exposed to increasing concentrations of imatinib. The established K562/IR subline could be maintained and passaged with 3 μM imatinib.

**Figure 1:**
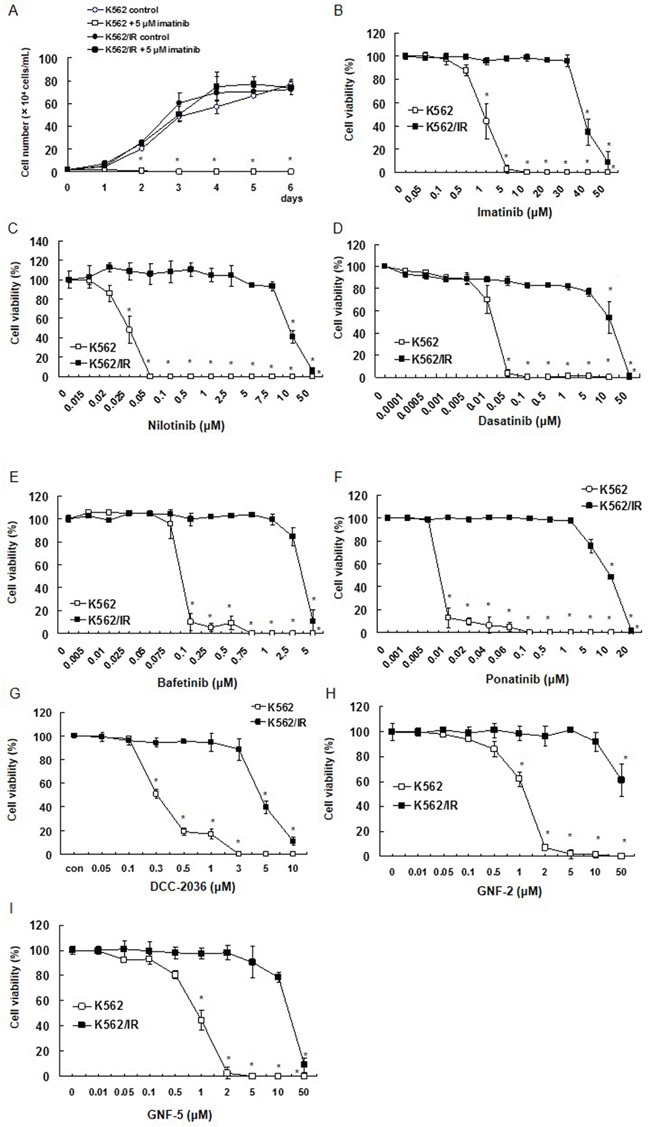
Establishment of K562/IR cells and their growth curves with imatinib treatment The effect of various BCR-ABL1 TKIs on cell survival/proliferation was determined using the trypan blue dye exclusion assay. **(A)** K562 and K562/IR cells were exposed to the indicated concentrations of imatinib. After incubation for 1, 2, 3, 4, 5, or 6 days, the number of viable cells was counted by trypan blue staining. These results are representative of 5 independent experiments. *p < 0.01 vs. untreated K562 cells as assessed with Dunnett's test. **(B-G)** Cell viability of K562/IR cells and their parental cell lines after exposure to different concentrations of **(B)** imatinib, **(C)** nilotinib, **(D)** dasatinib, **(E)** bafetinib, **(F)** ponatinib, **(G)** DCC-2036, **(H)** GNF-2, and **(I)** GNF-5 for 72 h. These results are representative of 5 independent experiments. *p < 0.01 vs. untreated K562 cells as assessed with Dunnett's test.

No significant difference in the growth of K562 cells was observed compared to K562/IR cells. Furthermore, imatinib did not suppress cell proliferation in K562/IR cells (Figure 1A). Viability of the K562/IR and parental K562 cells was also examined by determining the 50% inhibition concentration (IC50) for imatinib and other TKIs, including nilotinib, dasatinib, bafetinib, ponatinib, DCC-2036, GNF-2, and GNF-5. (Figure 1B-1I). We found that the resistant K562/IR cells displayed a significantly higher IC50 for viability compared to the parental K562 cells following exposure to all TKIs tested (Supplementary Figure 1). The resistant K562/IR cells showed no change in amount of proliferation compared to K562 cells. The resistance phenotype was stable, and the IC50 values and resistance indices for these drugs did not change over a 1-year period.

To validate these observations, we also established the imatinib-resistant subline KU812/IR. We performed similar growth curve analyses on the parental KU812 cells and the resistant KU812/IR subline following treatment with TKIs, which included imatinib, dasatinib, ponatinib, DCC-2036, and GNF-5. As with the K562/IR cell line, we found that the resistant KU812/IR cells displayed a significantly higher IC50 for viability compared to the parental KU812 cells following exposure to all TKIs tested (Supplementary Figure 2).

Next, we investigated whether the ABL1 gene was mutated in K562/IR cells. For K562/IR cells, polymerase chain reaction (PCR)-single strand conformation polymorphism (SSCP) analysis did not identify any ABL1 gene mutations within the region encoding amino acids 45 to 524. This result was confirmed by the Invader Assay, which did not detect any ABL1 gene mutations at 25 representative sites for K562/IR cells (Supplementary Figure 3).

### *MET*, *WNT2*, *BRAF*, and *EZH2* gene amplification in K562/IR cells

To identify chromosomal divergence between the parental cell line and its derivative, we performed array-based comparative genomic hybridization (CGH) analyses. These analyses identified multiple genes that were amplified only in K562/IR cells, but not in K562 cells. Among these, we focused on four genes that were amplified in K562/IR cells: MET, a member of the receptor tyrosine kinase family; wingless-type MMTV integration site family member 2 (WNT2), a member of the WNT gene family; BRAF, a member of MAPK signaling cascade; and enhancer of zeste 2 polycomb repressive complex 2 subunit (EZH2), a member of the histone methyltransferase complex (Table 1). These factors promote tumorigenesis, tumor progression, and drug resistance [16–19]. Thus, they may be important factors in imatinib resistance.

**Table 1:** Identification of genes amplified in K562/IR cells compared with parental K562 cells

Chrosome name	Cytoband	Gene name	K562 cells		K562/IR cells		P value
			log2 ratio	copy number	log2 ratio	copy number	
Chr2	q37.1	TRPM8	0	2	0.286653	2.4	4.71E-10
Chr3	p25.2	RPL32, SNORA7A	0	2	0.419834	2.7	2.80E-18
Chr5	q31.3	ARHGAP26, NR3C1	0	2	0.362194	2.6	1.42E-68
Chr7	p14.1	TARP	0	2	0.307947	2.5	2.92E-15
	q31.1	GPR85	0	2	0.501182	2.8	1.09E-79
	q31.2-q32.3	CAV2, CAV1, **MET**, CAPZA2, ST7OT1, ST7, ST7OT4, ST7OT2, ST7OT3, **WNT2**, ASZ1, CFTR, CTTNBP2, NAA38, ANKRD7, PTPRZ1, AASS, FEZF1, LOC154860, CADPS2, RNF133, RNF148, TAS2R16, SLC13A1, IQUB, NDUFA5, ASB15, LMOD2, HYALP1, HYAL4, SPAM1, TMEM229A, GPR37, LOC154872, POT1, GRM8, MIR592, ZNF800, GCC1, ARF5, FSCN3, PAX4, SND1, C7orf54, LRRC4, MIR593, MIR129-1, LEP, CALU, CCDC136, COPG2, KLF14, MIR29A, MIR29B1, LOC646329, FLJ43663, MKLN1	0	2	0.370068	2.6	4.11E-99
	q31.32-q31.33	SPAM1, TMEM229A, GPR37, LOC154872	0	2	0.270921	2.4	3.71E-17
	q31.33	GRM8, MIR592	0	2	0.282276	2.4	1.53E-20
	q32.1-q32.2	COPG2	0	2	0.459217	2.7	2.43E-28
	q33-q34	CREB3L2, AKR1D1, TRIM24, SVOPL, ATP6V0A4, TMEM213, KLRG2, RAB19, MKRN1, DENND2A, ADCK2, LOC100134713, NDUFB2, **BRAF**	0	2	0.391611	2.6	6.79E-39
	q36.1	ABP1, ACTR3C	0	2	0.404335	2.6	1.34E-12
	q36.1	C7orf33, CUL1, **EZH2**	0	2	0.417787	2.7	1.34E-12
	q36.1	GALNT11, GALNTL5	0	2	0.425165	2.7	9.11E-10
	q36.1	GIMAP1, GIMAP1-GIMAP5, GIMAP2, GIMAP4, GIMAP5, GIMAP6, GIMAP7, GIMAP8, KCNH2, LOC100128542, LOC285972, LOC728743, LRRC61, NOS3, RARRES2, REPIN, TMEM176A, TMEM176B, ZNF775	0	2	0.404335	2.6	1.34E-12
	q36.1	MLL3	0	2	0.425165	2.7	9.11E-10
	q36.3	C7orf13, NCRNA00244, RNF32	0	2	0.37813	2.6	6.82E-10
Chr9	q33.2	FBXW2, LOC100288842	0	2	1.311082	5.0	3.48E-305
Chr11	q22.3	CWF19L2	0	2	0.765187	3.4	2.72E-11
	q22.3	RDX	0	2	0.289343	2.4	1.22E-10

Values are log2 ratios.

**Figure 2:**
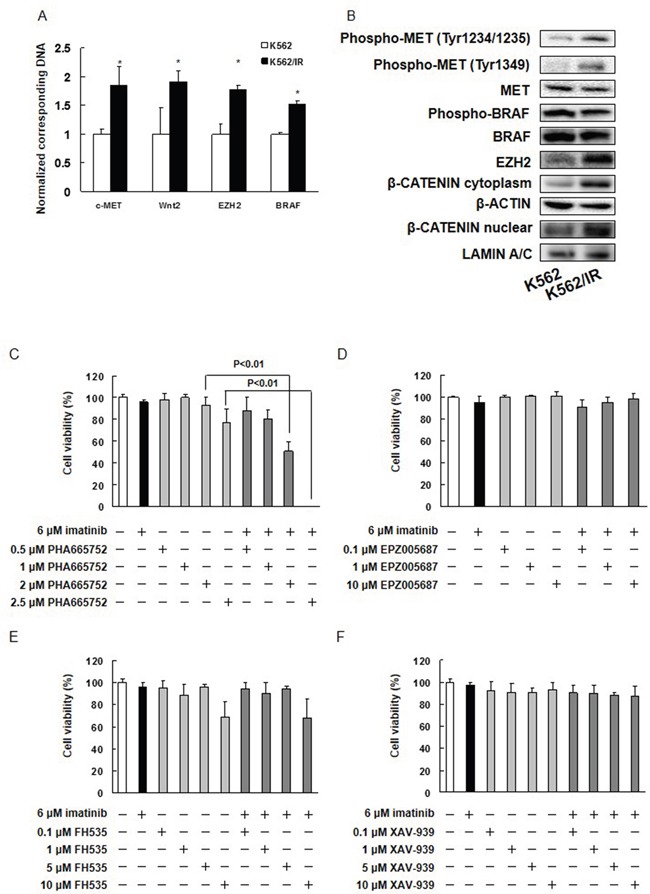
Inhibition of MET eliminates imatinib resistance in K562/IR cells **(A)** Expression of *c-MET*, *WNT2*, *EZH2*, and *BRAF* in parental and K562/IR cells. Genomic DNA was extracted, and *c-MET, WNT2, EZH2*, and *BRAF* levels were determined by real time PCR. The results are expressed as the test:control ratio after normalization using *GAPDH*. The results are representative of 5 independent experiments. *p < 0.01 vs. K562 cells as assessed by Dunnett's test. **(B)** Western blotting analysis. Samples of total cell lysates were separated by SDS-PAGE, transferred to polyvinylidene fluoride membranes, and incubated with primary antibodies against phospho-MET (Tyr1234/1235), phospho-MET (Tyr1349), phospho-BRAF (Ser445), EZH2, β-CATENIN, MET, BRAF, β-ACTIN, and LAMIN and then with a horseradish peroxidase-conjugate as the secondary antibody. **(C-F)** K562/IR cells were exposed to the indicated concentrations of imatinib, PHA665752, EPZ005687, FH535, or XAV-939. After incubation for 72 h, the number of dead cells was counted by trypan blue staining. The results are representative of 5 independent experiments. *p < 0.01 vs. untreated K562/IR cells (analysis of variance with Dunnett's test).

Next, we confirmed the CGH analyses by validating that expression of *MET, WNT2, BRAF*, and *EZH2* increased in K562/IR cells using real time PCR (Figure 2A). Lysates of the parental and derivative cells were also assayed by Western blotting. A dramatic increase in expression of EZH2, phospho-MET (Tyr1234/1235), and phospho-MET (Tyr1349) was observed in K562/IR cells relative to K562 cells, in addition to an increase in nuclear and cytoplasmic localization of β-CATENIN (Figure 2B). In contrast, expression levels of MET, phospho-BRAF, BRAF, phospho-BCR-ABL1, BCR-ABL1, phospho-SRC, SRC, phospho-FYN, FYN, phospho-LYN, LYN, phospho-YES, phospho-LCK, phospho-FGR, phospho-BLK, and phospho-HCK in parental and K562/IR cells were similar (Figure 2B, Supplementary Figure 4). We have also found MET activation in KU812/IR cells (Supplementary Figure 5A). Next, we investigated potential mutations in MET by qBiomarker. Somatic mutation PCR arrays in K562 and K562/IR cells. Surprisingly, the K562/IR cells did harbor the MET mutation Y1248C (Supplementary Figure 6). MET^Y1248C^ protein is very strongly activating. It promotes focus formation *in vitro*, and accelerates tumor formation in mice [20]. Thus, this type of mutation may be an important factor in conferring imatinib resistance.

### MET inhibition overcame imatinib resistance in K562/IR cells

We next treated K562/IR cells with small molecule antagonists of MET (PHA665752), EZH2 (EPZ005687), or β-CATENIN (FH535 and XAV-939) in order to determine whether imatinib sensitivity could be restored in K562/IR cells. We found that the combination of the MET inhibitor PHA665752 and imatinib induced cell death in K562/IR cells (Figure 2C). In addition, combining PHA665752 with nilotinib, dasatinib, bafetinib, ponatinib, GNF-2, or GNF-5 overcame the resistance of K562/IR cells to these drugs (Supplementary Figure 7A-7F). Other MET inhibitors, such as JNJ-38877605, EMD-1214063, foretinib, or crizotinib likewise restored imatinib sensitivity in K562/IR cells (Supplementary Figure 7G-7J). Moreover, co-treatment of PHA665752 or crizotinib with imatinib overcame the imatinib resistance in KU812/IR cells (Supplementary Figure 5B and 5C). Furthermore, PHA665752 and crizotinib induced cell death in high MET activation cell lines, such as HT29, DLD-1, and LoVo cells, but did not affect cell viability in low MET activation cell lines such as colo-205 (Supplementary Figure 8). In contrast, treatment of K562/IR cells with EPZ005687, FH535, or XAV-939 did not affect imatinib resistance (Figure 2D-2F and Supplementary Figure 9A). These data confirmed a central role for MET in the establishment of imatinib resistance.

### ERK1/2 and JNK are constitutively activated by MET, and inhibitors of these proteins restore imatinib sensitivity in K562/IR cells

**Figure 3:**
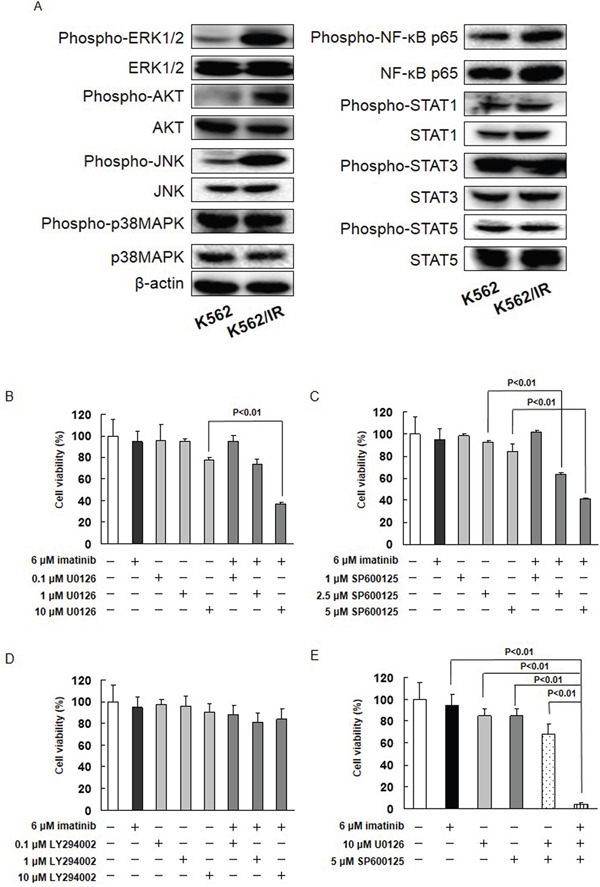
The MET/ERK and MET/JNK pathways contribute imatinib resistance **(A)** The cytoplasmic fractions of cells were extracted and subjected to SDS-PAGE/immunoblotting with anti-phospho-ERK1/2, anti-phospho-AKT, anti-phospho-JNK, anti-phospho-p38 MAPK, anti-phospho-NF-κB p65, anti-phospho-STAT1, anti-phospho-STAT3, anti-phospho-STAT5, anti-ERK1/2, anti-AKT, anti-JNK, anti-p38 MAPK, anti- NF-κB p65, anti-STAT1, anti-STAT3, and anti-STAT5 antibodies. Anti-β-ACTIN antibody was used as internal standards. **(B-E)** K562/IR cells were exposed to the indicated concentrations of imatinib, U0126, SP600125, or LY294002. After incubation for 72 h, the number of dead cells was counted by trypan blue staining. The results are representative of 5 independent experiments. *p < 0.01 vs. untreated cells as assessed with Dunnett's test.

Thus far, our data indicate that imatinib resistance is achieved via activation of MET. We therefore investigated the activity of downstream effectors of MET signaling in K562/IR cells. The expression of phosphorylated ERK1/2, JNK, and AKT was higher in K562/IR and KU812/IR cells compared to K562 and KU812 cells, respectively (Figure 3A, Supplementary Figure 5A). The expression of phosphorylated STAT1, STAT3, STAT5, NF-κB, and p38 MAPK, however, did not differ between the parental and drug-resistant K562/IR cells (Figure 3A).

**Figure 4:**
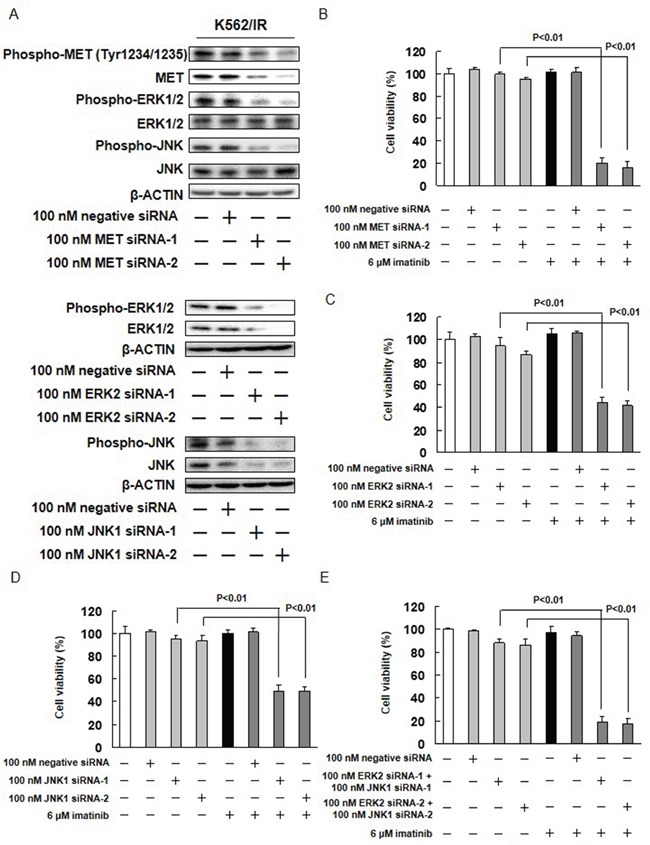
Effect of MET, ERK2, and JNK1 siRNAs on imatinib resistance **(A)** K562/IR cells were treated with MET siRNA, ERK2 siRNA, JNK1 siRNA, or a negative control siRNA for 1 day. Control cells were treated with PBS and cultured in serum-containing medium for 3 days. phospho-MET, anti-phospho-ERK1/2, anti-phospho-JNK, anti-MET, anti-ERK1/2, and anti-JNK antibodies. Anti-β-ACTIN antibody was used as internal standards. **(B-E)** K562/IR cells were exposed to the indicated concentrations of MET siRNA, ERK siRNA, or JNK siRNA. After incubation for 72 h, the number of dead cells was counted by trypan blue staining. The results are representative of 5 independent experiments. *p < 0.01 vs. untreated K562/IR cells as assessed with Dunnett's test.

The potential role of ERK1/2, JNK, and AKT in the imatinib resistance exhibited by K562/IR cells was further investigated. The effect of imatinib alone and in combination with MEK1/2, JNK, or PI3K inhibitors (U0126, SP600125, or LY294002, respectively) on the viability of K562/IR cells was assessed. The combination of 10 μM U0126 with imatinib enhanced the sensitivity of K562/IR cells to imatinib (Figure 3B and Supplementary Figure 9B). Furthermore, the addition of 2.5 or 5 μM SP600125 to imatinib restored the sensitivity of K562/IR cells to imatinib (Figure 3C, Supplementary Figure 9B). However, LY294002 did not affect the sensitivity of K562/IR cells to imatinib (Figure 3D and Supplementary Figure 9B). Treatment with both of these inhibitors (10 μM U0126 and 5 μM SP600125) in combination profoundly overcame the imatinib resistance of the K562/IR cells (Figure 3E). In addition, the combination of MET siRNA, ERK siRNA, or JNK siRNA with imatinib enhanced the sensitivity of K562/IR cells to imatinib (Figure 4).

**Figure 5:**
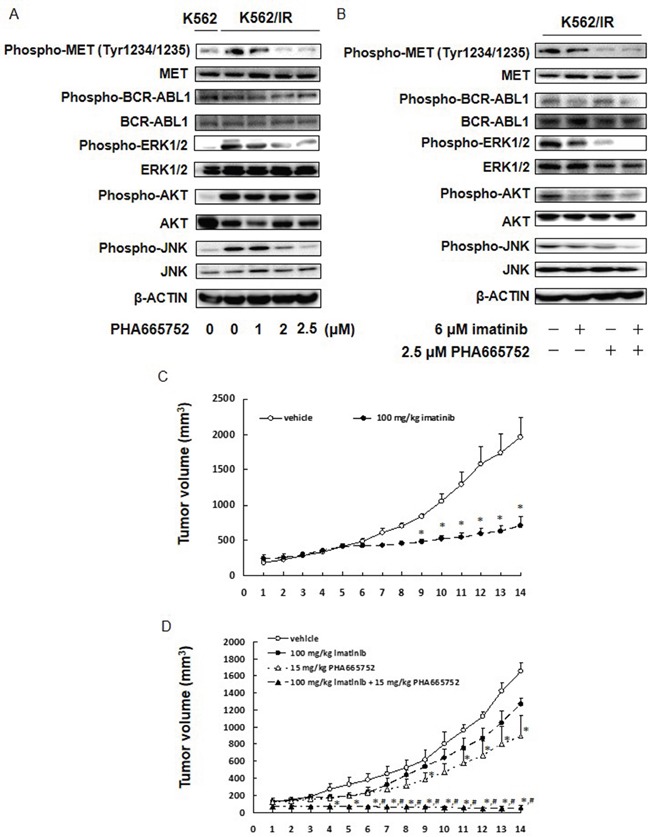
MET inhibitor inhibits the ERK and JNK activation, and combined treatment of MET inhibitor and imatinib significantly suppressed tumor growth of K562/IR cells *in vivo* **(A, B)** K562/IR cells were exposed to the indicated concentrations of imatinib or PHA665752. After incubation for 48 h, the cytoplasmic fractions were extracted and then subjected to SDS-PAGE/immunoblotting with anti-phospho-MET, anti-phospho-ABL1, anti-phospho-ERK1/2, anti-phospho-AKT, anti-phospho-JNK, anti-MET, anti-ABL1, anti-ERK1/2, anti-AKT, and anti-JNK antibodies. Anti-β-ACTIN antibody was used as internal standards. **(C)** K562 or **(D)** K562/IR xenograft model. On day 0, mice were treated with imatinib and/or PHA665752. Imatinib was administered orally (p.o.) at 100 mg/kg daily over a period of 2 weeks; n = 5 for each group. PHA665752 was administered intraperitoneally (i.p.) at 15 mg/kg daily over a period of 2 weeks; n = 5 for each group. Tumor volumes are presented as means ± S.E.M. *p < 0.01 vs. controls, and ^#^p < 0.01; 100 mg/kg imatinib + 15 mg/kg PHA665752 vs. 100 mg/kg imatinib (ANOVA with Dunnett's test).

Finally, we sought to validate that PHA665752 indeed inhibited the MET/ERK and MET/JNK pathways in K562/IR and KU812/IR cells. As expected, treatment with PHA665752 reduced the expression of phospho-MET (Tyr1234/1235), phospho-ERK1/2, and phospho-JNK, but not phospho-BCR-ABL1 or phospho-AKT (Figure 5A and Supplementary Figure 10). In addition, the combination of PHA665752 and imatinib significantly inhibited ERK1/2 and JNK activation (Figure 5B, Supplementary Figure 10). Although treatment with imatinib suppressed the activation of BCR-ABL1 and AKT, the combination of PHA665752 and imatinib did not enhance BCR-ABL1 and AKT suppression (Figure 5B, Supplementary Figure 10). While imatinib suppressed the tumor growth of K562 cells *in vivo*, it did not affect the tumor growth of K562/IR cells (Figure 5C-5D). In contrast, combined treatment of imatinib and PHA665752 significantly inhibited the tumor growth of K562/IR cells *in vivo* (Figure 5D). Cumulatively, these results indicate that the MET/ERK and MET/JNK pathways may play a critical role in the mechanism of imatinib resistance in K562/IR cells.

## DISCUSSION

In this study, we found that K562/IR and KU812/IR cells exhibited strong resistance to many BCR-ABL1 TKIs. The resistance phenotype was stable, and the IC50 values and resistance indices for these drugs did not change over a 1-year period.

Over 100 BCR-ABL1 kinase domain point mutations have been linked to clinical imatinib resistance [21], and resistance profiles for newer BCR-ABL1 TKIs mainly comprise subsets of these mutations. Each mutation has been implicated in resistance to one or more of the following TKIs: imatinib, nilotinib, dasatinib, and ponatinib [22–24]. The key residues that became mutataed in native BCR-ABL1 are M244, L248, Q250, Q252, Y253, E255, E279, F311, T315, F317, M351, M351, F359, V379, L387, H396, S417, E459, and F489. In addition, combined mutations of E255V/T315I, T315I/F359C, Y253H/T315I, T315I/H396R, or T315I/E453K can cause ponatinib resistance clinically [24]. In our current study, K562/IR cells did not have any ABL1 gene mutations. This observation suggested that the resistance of K562/IR cells depends on a BCR-ABL1-independent mechanism.

To gain insights into the mechanisms of BCR-ABL1 TKI resistance, we carried out an array CGH to identify genes differentially expressed in parental versus K562/IR cells. Of the potential resistance genes identified in the present study, we focused our attention on MET, WNT2, BRAF, and EZH2. We observed that expression levels of EZH2, phospho-MET, and β-CATENIN (a signaling molecule downstream of WNT2) were elevated in K562/IR cells. However, the expression and activation of BRAF, BCR-ABL1, SRC, LYN, FYN, YES, LCK, FGR, BLK, and HCK did not increase. Moreover, activation of MET in KU812/IR cells was increased compared to the parental KU812 cells. We showed that inhibition of MET activity by the MET inhibitor, PHA665752, resensitized K562/IR cells to imatinib, while treatment with the EZH2 inhibitor, EPZ005687, or the WNT/β-CATENIN signaling inhibitors, FH535 and XAV-939, did not. In addition, PHA665752 reversed the resistance of K562/IR cells to various other BCR-ABL1 TKIs. Moreover, numerous MET inhibitors resensitized K562/IR cells to imatinib, and PHA665752 and crizotinib restored sensitivity to imatinib in KU812/IR cells. The ability of MET to induce proliferation, protect from apoptosis, and enhance angiogenesis and cell motility suggested that it may contribute to tumorigenesis. In addition, activation of MET has been observed in response to treatment with EGFR TKIs in lung cancers and anti-EGFR antibodies in colorectal cancers [25]. We found the MET mutation Y1248C in K562/IR cells, and MET^Y1248C^ strongly activates MET and promotes tumor formation [20]. Mutants of MET are known to promote tumorigenesis, tumor progression, and drug resistance [17]. Our findings favor an important role for MET in resistance to BCR-ABL1 TKIs.

Activation of MET promotes the phosphorylation of ERK, AKT, NF-κB, p38 MAPK, JNK, STAT1, STAT3, and STAT5 [17]. The activation of these signaling molecules by MET plays a key role not only in the development of many cancers, but also in drug resistance mechanisms implemented by tumor cells. We found that the expression of phosphorylated ERK1/2, AKT, and JNK was elevated in K562/IR cells, but the activation of NF-κB, p38 MAPK, STAT1, STAT3, and STAT5 was not. We also observed that U0126 and SP600125 partially resensitized K562/IR cells to imatinib, while LY294002, a PI3K inhibitor, did not affect imatinib resistance. In addition, the combination of U126 and SP600125 with imatinib strongly induced cell death, as did various MET inhibitors. Furthermore, co-treatment of MET, ERK, or JNK siRNA with imatinib induced cell death in K562/IR cells. Treatment with PHA665752 in K562/IR and KU812/IR cells inhibited MET, ERK1/2, and JNK activation, but did not affect AKT activation. Moreover, combined treatment of K562/IR and KU812/IR cells with imatinib and PHA665752 inhibited ERK1/2 and JNK activation, and had a stronger effect on the K562/IR and KU812/IR cells than either agent alone. In addition, we found that combined treatment of imatinib and PHA665752 significantly inhibited tumor growth of K562/IR cells *in vivo*. Combined treatment with imatinib and trametinib, a MEK inhibitor, causes cell death, and prolongs survival in mouse models of BCR-ABL1-independent imatinib-resistant CML [26]. Parker et al. suggested that nilotinib synergizes with MEK inhibitors to kill drug-resistant CML cells, and blocks tumor growth in mice [27]. Cui et al. reported that basal JNK activity is essential for survival and proliferation of T-cell acute lymphoma [28]. These findings suggest that activation of ERK1/2 and JNK may play important roles in imatinib resistance. Moreover, we showed that activation of JNK is involved in mutation-independent BCR-ABL1 TKI resistance. In patients with imatinib-resistant CML, activation of ERK1/2 is correlated with BCR-ABL1-independent imatinib resistance [29]. Although activation of MET and JNK is not reported to be associated with BCR-ABL1-independent imatinib resistance in CML patients, there are indications that ABL1 interacts with oncogenic MET [30]. Furthermore, increased JNK expression and JNK signaling in primary CML cells are implicated in BCR-ABL1-induced leukemogenesis [31]. Collectively, these results indicate that activation of the MET/ERK and MET/JNK pathways may be involved in BCR-ABL1 TKI resistance, and inhibition of the MET/ERK and MET/JNK pathways can resensitize resistant CML cells to BCR-ABL1 TKIs.

Previous studies indicated that overexpression and/or activation of BCR-ABL1, Lyn, Hck, and Fyn is involved in imatinib resistance in K562 cells [12, 32–34]. It has also been reported that P-glycoprotein overexpression or down-regulation of p21^cip1^ expression confers resistance to imatinib in K562 cells [35–37]. High levels of activation of STAT3 or STAT5 enhance resistance to imatinib in K562 cells [38, 39]. In this study, we found that expression and activation of BCR-ABL1, SRC, LYN, FYN, YES, LCK, FGR, BLK, HCK, STAT3, and STAT5 did not increase in K562/IR cells. In addition, although the expression of P-glycoprotein was higher in K562/IR cells than in K562 cells, treatment of MDR1 siRNA did not enhance imatinib sensitivity in K562/IR cells (data not shown). Furthermore, no substantial difference in the expression level of p21^cip1^ was observed between K562/IR and K562 cells (data not shown). These results indicate that the activation of MET may contribute to imatinib resistance through mechanisms independent of these factors.

We observed that the Y1248C mutation caused constitutive activation of MET in K562/IR cells. It is tempting to speculate that during the selection process initiated by low concentrations of BCR-ABL1 TKIs, cells with the Y1248C mutation benefit from the constitutive activation of MET because it counteracts the effects of BCR-ABL1 inhibition. This might allow cells to resist higher doses of imatinib preferentially, leading to the selection of clones with high levels of MET activation. Altogether, our findings show that increased MET activity is an important determinant of imatinib resistance in BCR-ABL1 TKI-resistant cells.

In conclusion, gene expression profiling identified several new genes associated with resistance to BCR-ABL1 TKIs. Our results show an important function for MET in resistance to imatinib and other BCR-ABL1 TKIs. Further studies are needed to better define the roles these genes play in CML progression and resistance to BCR-ABL1 TKIs. Our findings offer new and significant insights concerning the mechanisms of resistance of CML cell lines, and reveal new proteins potentially involved in resistance to BCR-ABL1 TKIs. This may have useful implications for the establishment of future therapies for CML and other hematopoietic malignancies.

## MATERIALS AND METHODS

### Materials

Imatinib, nilotinib, dasatinib, bafetinib, ponatinib, DCC-2036, GNF-2, GNF-5, EPZ005687, XAV-939, and FH535 were purchased from Selleckchem (Houston, TX, USA). PHA665752 was purchased from MedChemExpress, LLC (Princeton, NJ, USA). U0126, LY294002, and SP600125 were purchased form Wako (Tokyo, Japan). These reagents were dissolved in dimethyl sulfoxide and diluted in phosphate-buffed saline (PBS; 0.05 M, pH 7.4), filtrated through syringe filters (0.45 μm, IWAKI GLASS, Tokyo, Japan), and used for various assays described below.

### Cell culture

The CML cell line K562 was obtained from Health Science Research Resources Bank (Osaka, Japan). The cells were cultured in RPMI1640 medium (Sigma) supplemented with 10% fetal calf serum (Gibco, Carlsbad, CA, USA), 100 μg/ml penicillin (Gibco), 100 U/ml streptomycin (Gibco), and 25 mM HEPES (pH 7.4; Wako) in an atmosphere containing 5% CO2.

### Establishment of acquired resistance to imatinib

Over a period of 6 months, K562 cells were grown in culture and continuously exposed to increasing concentrations of imatinib. Commencing at the IC50 of imatinib for K562 cells, the exposure dose was progressively doubled every 10 to 14 days until 7-8 dose doublings had been achieved. The subline of resistant cells was then maintained in continuous culture with 3 μM of imatinib for 2 months; this was the highest dose that still allowed cellular proliferation. The resistant phenotype has been stable for at least 1 year under drug-free conditions.

### Trypan blue dye exclusion assay

The effect of various anti-cancer drugs on cell survival/proliferation was determined using the trypan blue dye exclusion assay. Prior to each experiment, cells (3 × 103 cells/well) were plated onto 96-well plates. After culturing for 24 h, the cells were exposed to anti-cancer drugs for various times. Equal volumes of cell suspension and 0.4% trypan blue solution were mixed gently, loaded into a hemocytometer, and the viable cells (unstained) and dead cells (stained blue) were counted. Each experiment was performed in triplicate. Results are reported from an average of at least 5 independent experiments.

### Array-CGH

The Genome-wide Human SNP Array 6.0 (Affymetrix, Santa Clara, CA) was used to perform array-CGH on genomic DNA from each of the cell lines, in accordance with the manufacturer's instructions. A total of 250 ng of genomic DNA was digested with the restriction enzymes Nsp I and Sty I in independent parallel reactions (SNP6.0), ligated to the adaptor, and amplified using PCR with a universal primer and TITANIUM Taq DNA Polymerase (Clontech). The PCR products were quantified, fragmented, end-labeled, and hybridized onto a Genome-wide Human SNP Array 6.0. After washing and staining in Fluidics Station 450 (Affymetrix), the arrays were scanned to generate CEL files using the GeneArray Scanner 3000 and GeneChip Operating Software ver.1.4. In the array-CGH analysis, sample-specific changes in copy number were analyzed using Partek Genomic Suite 6.4 software (Partek Inc., St. Louis, MO).

### Quantitative genomic PCR

Genomic DNA from cultured cells was extracted using a Nucleo Spin Tissue kit (Takara Biomedical, Siga, Japan) according to the manufacturer's protocol. Genomic DNA was subjected to quantitative real time PCR using SYBR Premix Ex Taq (Takara Biomedical) and the Thermal Cycler Dice Real Time system (Takara Biomedical) in a 96-well plate according to the manufacturer's instructions. The PCR conditions for *GAPDH*, *c-MET*, *WNT2*, *EZH2*, and *BRAF* were 94°C for 2 min, followed by 40 cycles of 94°C for 0.5 min, 50°C for 0.5 min, and 72°C for 0.5 min. The following primers were used: *c-MET*, 5′- CCA CAA GCC CTG CTA ATC TG -3′ (5′-primer) and 5′- CCA GTG TGT AGC CAT TTT GG -3′ (3′-primer); *WNT2*, 5′- AGT GGC AAA GGT TGT CTG AAA -3′ (5′-primer) and 5′- CCC TGG TGA TGG CAA ATA CAA C -3′ (3′-primer); *EZH2*, 5′- TTA GAT GGC CAG CAA CAC AG -3′ (5′-primer) and 5′- GGC ATC AGC CTG GCT GTA TC -3′ (3′-primer); *BRAF*, 5′- AAG GGG ATC TCT TCC TGT ATC C -3′ (5′-primer) and 5′- GCC ACT TTC CCT TGT AGA CT -3′ (3′-primer); and *GAPDH*, 5′-GAC ATC AAG AAG GTG GTG AA-3′ (5′-primer) and 5′- CCA GCC ACA TAC CAG GAA AT-3′ (3′-primer). As an internal control for each sample, *GAPDH* was used for standardization. Cycle threshold (Ct) values were recorded, and the normalized expression of each gene in control versus TKI-resistant cells was calculated using the 2–ΔΔCt method.

### Western blotting

The cytoplasm and nuclear fractions of K562 and K562/IR cells were extracted with the ProteoExtract Subcellular Proteome Extraction Kit (Calbiochem, San Diego, CA, USA). The protein content in the cell lysates was determined using a BCA protein-assay kit. The extracts (40 μg of protein) were fractionated on polyacrylamide-SDS gels and transferred to polyvinylidene fluoride (PVDF) membranes (Amersham, Newark, NJ, USA). The membranes were blocked with a solution containing 3% skim milk and incubated overnight at 4°C with each of the following antibodies: anti-phospho-MET (Tyr1234/1235) antibody, anti-phospho-MET (Tyr1349) antibody, anti-phospho-BRAF (Ser445) antibody, anti-phospho-STAT1 (Tyr701) antibody, anti-phospho-STAT3 (Tyr705) antibody, anti-phospho-STAT5 (Tyr694) antibody, anti-phospho-ERK1/2 (Thr202/Tyr204) antibody, anti-phospho-AKT (Ser473) antibody, anti-phospho-JNK (Thr183/Tyr185) antibody, anti-phospho-NF-κB p65 (Ser536) antibody, anti-phospho-p38 MAPK (Thr180/Tyr182) antibody, anti-EZH2 antibody, anti-β-catenin antibody, anti-MET antibody, anti-BRAF antibody, anti-STAT1 antibody, anti-STAT3 antibody, anti-STAT5 antibody, anti-ERK1/2 antibody, anti-AKT antibody, anti-JNK antibody, anti-NF-κB antibody, anti-p38 MAPK antibody (Cell Signaling Technology, Beverly, MA, USA), anti-LAMIN A/C antibody (Santa Cruz Biotechnologies, CA, USA), and anti-β-ACTIN antibody (Sigma). Subsequently, the membranes were incubated with horseradish peroxidase-coupled anti-rabbit IgG sheep antibodies (Amersham) for 1 h at room temperature. The reactive proteins were visualized using ECL-plus (Amersham) according to the manufacturer's instructions.

### RNA interface

The double-stranded small interfering RNAs (siRNAs) targeting MET (HSS106477 and HSS106478), ERK2 (VHS40312 and VHS40318), and JNK1 (VHS40722 and VHS40724) were synthesized and purified by Invitrogen (Carlsbad, CA, USA). Stealth^TM^ RNAi negative control duplex (low GC content) (Invitrogen) was used as a negative control. Transfection of siRNAs was performed according to the manufacturer's protocol by using the Lipofectamine^TM^ 2000 reagent (Invitrogen). Briefly, 4 μl of 20-μM siRNA was mixed with 200 μl of Opti-minimum essential medium (MEM^®^). Lipofectamine^TM^ 2000 (4 μl) was diluted in 200 μl of Opti-MEM^®^ and incubated at room temperature for 5 min. After incubation, the diluted Lipofectamine^TM^ 2000 was mixed with the diluted siRNA and further incubated for 20 min at room temperature. In total, 400 μl of the siRNA-Lipofectamine^TM^ 2000 complex was applied to each well containing cultured K562/IR cells at approximately 50–70% confluence in 6-well microplates.

### Xenograft mouse model of K562 and K562/IR cells

To induce a CML xenograft model, K562 and K562/IR cells were grown to 80% confluence. Cell viability was confirmed by trypan blue exclusion. Suspensions consisting of single cells with >90% viability were injected subcutaneously (s.c.) as a bolus of 1 × 10^6^ cells in 50 μL of phosphate buffered saline (PBS) into the left flank of each male CB17.Cg-PrkdcscidLystbg-J/CrlCrlj mouse (Charles River, Yokohama, Japan). Treatment began 1 week after inoculation, with mice bearing subcutaneous tumors of approximately 100 mm^3^ size at the start of the treatment. Tumors were measured daily with a caliper square and their volumes were calculated using the formula (*a*×*b*^2^)/2, where *a* and *b* are the larger and smaller diameters, respectively. Animal studies were performed in accordance with the Recommendations for Handling of Laboratory Animals for Biomedical Research compiled by the Committee on Safety and Ethical Handling Regulations for Laboratory Animal Experiments, Kinki University. The ethical procedures followed met the requirements of the United Kingdom Coordinating Committee for Cancer Research (UKCCCR) guidelines (2010).

### Statistical analysis

All results are expressed as means and standard deviations of several independent experiments. Multiple comparisons of the data were made using ANOVA with Dunnett's test. P values less than 5% were regarded as significant.

## SUPPLEMENTARY MATERIALS FIGURES AND TABLES



## Abbreviations

ALL: acute lymphoblastic leukemia
BCR-ABL1: breakpoint cluster region-abelson 1
CGH: comparative genomic hybridization
CML: Chronic myeloid leukemia
EGFR: epidermal growth factor receptor
ERK1/2: extracellular signal-regulated kinase
EZH2: enhancer of zeste 2 polycomb repressive complex 2 subunit
JAK: janus kinase
JNK: c-Jun N-terminal kinase
MAPK: mitogen-activated protein kinase
PCR: polymerase chain reaction
PI3K: phosphatidylinositide-3 kinase
SSCP: single strand conformation polymorphism
STAT: signal transduction and transcription
TKI: tyrosine kinase inhibitor
WNT2: wingless-type MMTV integration site family member 2.
