# Supplementary material for: Contributions of MET activation to BCR-ABL1 tyrosine kinase inhibitor resistance in chronic myeloid leukemia cells
Source: Oncotarget. 2017 Mar 17;8(24):38717–30. doi: 10.18632/oncotarget.16314 (PMC5503566; doi:10.18632/oncotarget.16314)
Supplement: Supplementary file 1 [file oncotarget-08-38717-s001.pdf]

Contributions of MET activation to BCR-ABL1 tyrosine kinase inhibitor resistance in chronic myeloid leukemia cells

SUPPLEMENTARY MATERIALS

1. Yamamoto M, Kakihana K, Ohashi K, Yamaguchi T, Tadokoro K, Akiyama H, Sakamaki H. Serial monitoring of T315I BCR-ABL mutation by Invader assay combined with RT-PCR. Int J Hematol 2009; 89: 482-488.

Cell line	IC_{50} (μM)							
	imatinib	nilotinib	dasatinib	bafetinib	ponatinib	DCC-2036	GNF-2	GNF-5
K562	0.815	0.031	0.012	0.091	0.008	0.358	1.322	0.721
K562/IR	31.926	9.712	11.059	2.809	5.702	4.082	66.764	19.791
IC_{50}-fold increase	39.173	313.29	921.58	30.868	712.75	11.402	50.482	27.456

Supplementary Figure 1: IC50 of various BCR-ABL kinase inhibitors for K562 and K562/IR cells. Cell viability was analyzed by Trypan blue dye exclusion assay. The IC50s were calculated by fitting the data to a logistic curve.

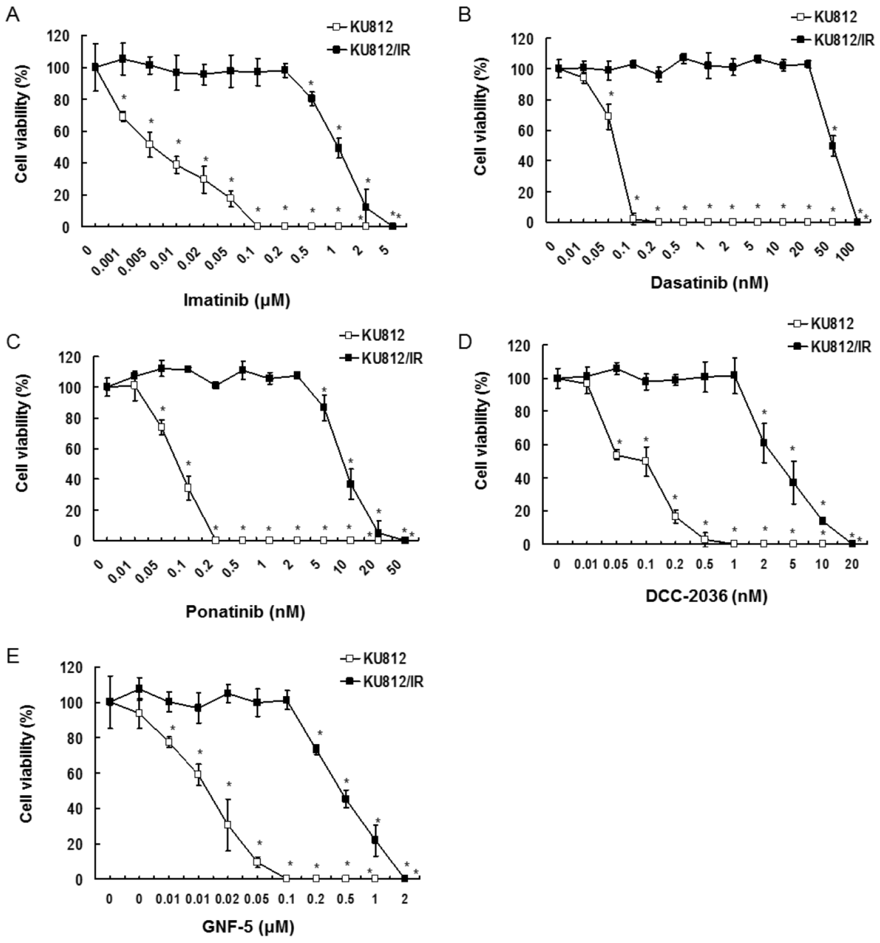

F

Cell line	IC_{50} (nM)				
	imatinib	dasatinib	ponatinib	DCC-2036	GNF-5
KU812	5.547	0.053	0.067	0.068	10.317
KU812/IR	942.7	36.919	7.201	3.689	434.81
IC_{50}-fold increase	169.95	702.03	107.21	54.143	42.143

Supplementary Figure 2: Establishment of KU812/IR cells and their cell viability with various BCR-ABL1 TKIs treatment. The effect of various BCR-ABL1 TKIs on cell survival was determined using the trypan blue dye exclusion assay. **(A-E)** Cell viability of KU812/IR cells and their parental cell lines after exposure to different concentrations of **(A)** imatinib, **(B)** dasatinib, **(C)** ponatinib, **(D)** DCC-2036, and **(E)** GNF-5 for 72 h. These results are representative of five independent experiments. *$p < 0.01$ vs. untreated KU812 cells as assessed with Dunnett's test. **(F)** The IC50s were calculated by fitting the data to a logistic curve.

Cell line	Kinase domain mutation								
	M244V	L248V	Q250E	Q252H	Y253F	E255K	E279K	F311L	T315I
K562/IR	N.D.	N.D.	N.D.	N.D.	N.D.	N.D.	N.D.	N.D.	N.D.

Cell line	Kinase domain mutation								
	F317L	M351T	F359I	V379I	L387M	H396R	S417Y	E459K	F489S
K562/IR	N.D.	N.D.	N.D.	N.D.	N.D.	N.D.	N.D.	N.D.	N.D.

Supplementary Figure 3: Detection of ABL1 gene mutation in K562/IR cells. The detection of 18 mutations (M244V, L248V, G250E, Q252H, Y253F, E255K, E279K, F311L, T315I, F317L, M351T, F359I, V379I, L387M, H396R, S417Y, E459K and F486S) in BCR-ABL1 KD was performed with PCR-Invader assay according to previous reports with minor modification [1]. Briefly, the primary probe and Invader oligo for detection of each mutation were designed with the Invader technology creator (TWT, Madison, WI, USA) and were based on the ABL1 (accession no. NM 005157) sequences. The Invader reactions were performed using 384-well plates with reagents contained in the Cleavase XI Invader core reagent kit (Amplified DNA) (TWT, Madison, WI, USA), 70 nM primary probes, 7 nM Invader oligo, and 10−2 dilution PCR amplicon which was denatured at 95°C for 5 min. Plates were incubated at 65°C in the fluorescence micro plate reader (FluoDia-T70; Otsuka Electronics, Osaka, Japan). Fluorescence values of FAM (carboxyfluorescein) (wavelength/bandwidth: excitation, 485/20 nm; emission, 530/25 nm) for mutation and RED (REDmond RED) (excitation, 560/20 nm; emission, 620/40 nm) for wild type were measured 30 min later. The fold-over-zero (FOZ) values were used to normalize the difference between measurements. The FOZ values were calculated by dividing the fluorescence value of sample by that of the negative control. The mutations were identified by calculation of the FOZ values.

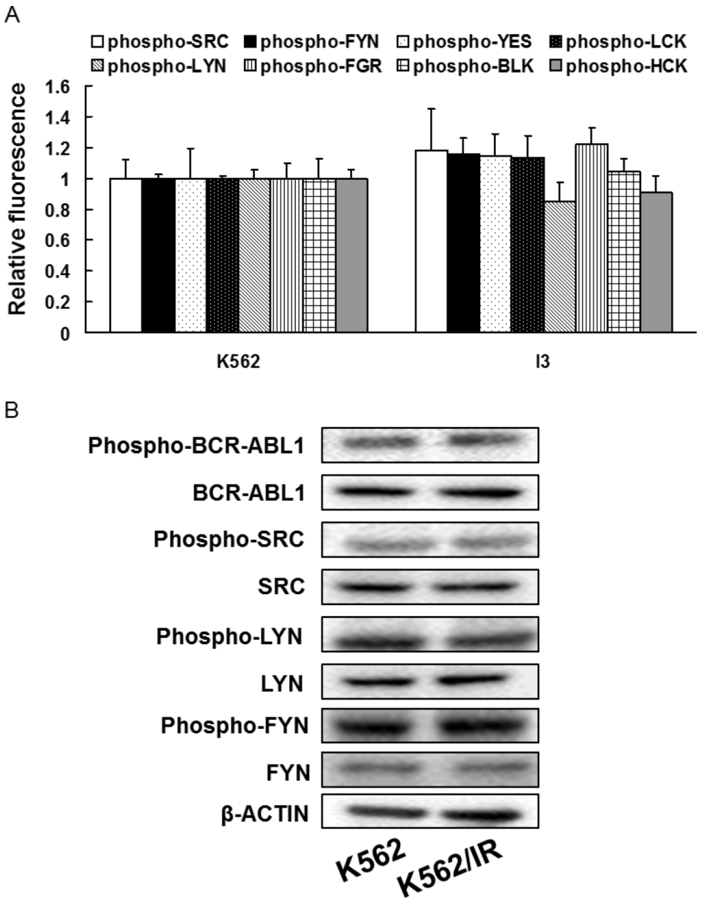

Supplementary Figure 4: Expression of BCR-ABL1, SRC, FYN, YES, LCK, LYN, FGR, BLK, and HCK in parental and K562/IR cells. **(A)** Cells were lysed and phosphorylation of Src family kinase protein were measured by Luminex assay. **(B)** Western blotting analysis: Samples of total cell lysates were separated by SDS-PAGE, transferred to polyvinylidene fluoride membranes, and incubated with primary antibodies against phospho-ABL1, phospho-SRC, phospho-LYN, phospho-FYN, ABL1, SRC, LYN, FYN, and β-ACTIN, and then with a horseradish peroxidase-conjugate as the secondary antibody.

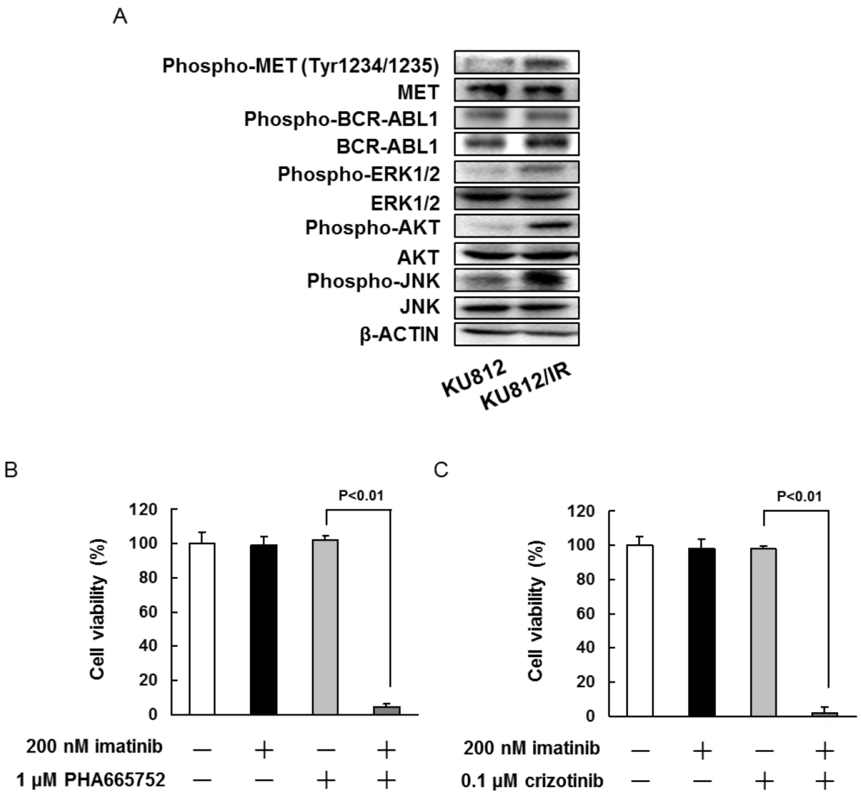

Supplementary Figure 5: Expression of MET, BCR-ABL1, ERK, AKT, and JNK in parental and KU812/IR cells. (A) Western blotting analysis: Samples of total cell lysates were separated by SDS-PAGE, transferred to polyvinylidene fluoride membranes, and incubated with primary antibodies against phospho-MET, phospho-ABL1, phospho-ERK, phospho-Akt, phospho-JNK, MET, ABL1, ERK, AKT, JNK, and β-ACTIN, and then with a horseradish peroxidase-conjugate as the secondary antibody. **(B)** KU812/IR cells were exposed to the indicated concentrations of imatinib, PHA665752, or crizotinib. After incubation for 72 h, the number of dead cells was counted by trypan blue staining. The results are representative of 5 independent experiments. *$p < 0.01$ vs. untreated KU812/IR cells (analysis of variance with Dunnett's test).

Cell line	MET mutation								
	E168D	N375S	L982-D1028del	R988C	T1010I	Y1248H	Y1248C	Y1253D	M1268T
K562	N.D.	N.D.	N.D.	N.D.	N.D.	N.D.	N.D.	N.D.	N.D.
K562/IR	N.D.	N.D.	N.D.	N.D.	N.D.	N.D.	+	N.D.	N.D.

Supplementary Figure 6: Detection of MET gene mutations in K562/IR cells. DNA was isolated using Nucleo Spin Tissue kit (Takara Biomedical) as per the supplier's protocol. We carried out genomic mutation analysis of the cohort using the SABiosciences qBiomarker Somatic Mutation PCR array (QIAGEN) for the c-MET pathway. Common mutation analysis was performed for the MET genes. All mutations were confirmed by direct sequencing of the DNA sample from K562/IR cells after an independent PCR reaction, and confirmed to be somatic by sequencing of the matched sample of K562 cells.

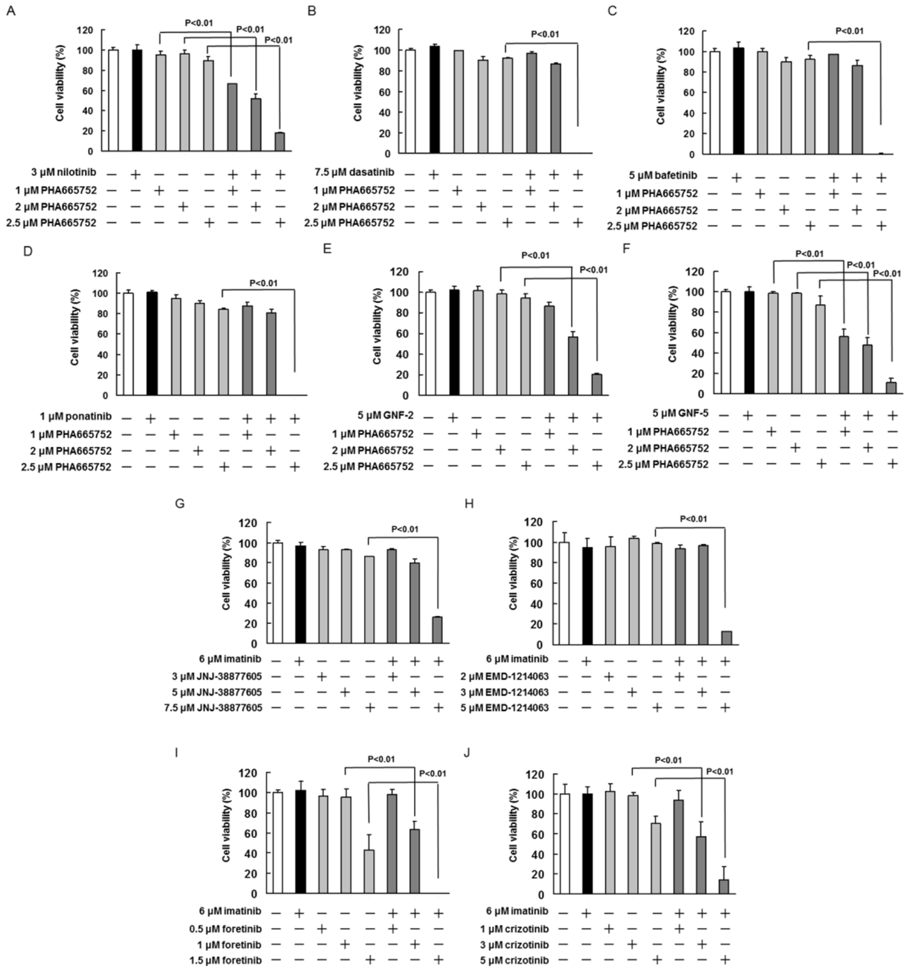

Supplementary Figure 7: MET inhibition overcomes the resistance to various BCR-ABL1 TKIs in K562/IR cells. (A-F) K562/IR cells were exposed to the indicated concentrations of PHA665752, nilotinib, dasatinib, bafetinib, ponatinib, GNF-2, or GNF-5. After incubation for 72 h, the number of dead cells was counted by trypan blue staining. The results are representative of 5 independent experiments. *p < 0.01 vs. untreated K562/IR cells as assessed with Dunnett's test. **(G-J)** K562/IR cells were exposed to the indicated concentrations of imatinib, JNJ-38877605, EMD-1214063, foretinib, or crizotinib. After incubation for 72 h, the number of dead cells was counted by trypan blue staining. The results are representative of 5 independent experiments. *p < 0.01 vs. untreated K562/IR cells as assessed with Dunnett's test.

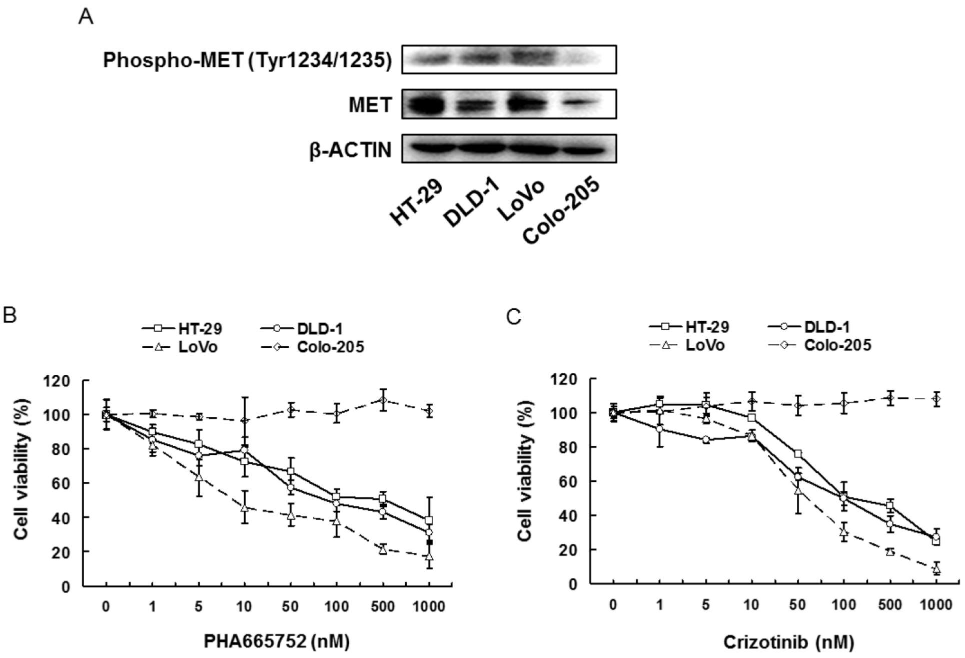

Supplementary Figure 8: PHA665752 and crizotinib iuduced cell death in MET high expression cancer cells than low expression cancer cells. **(A)** Western blotting analysis: Samples of total cell lysates were separated by SDS-PAGE, transferred to polyvinylidene fluoride membranes, and incubated with primary antibodies against phospho-MET, MET, and β-ACTIN, and then with a horseradish peroxidase-conjugate as the secondary antibody. **(B)** HT-29, DLD-1, LoVo, and Colo-205 cells were exposed to the indicated concentrations of **(B)** PHA665752 or **(C)** crizotinib. After incubation for 72 h, the number of dead cells was counted by trypan blue staining. The results are representative of 5 independent experiments.

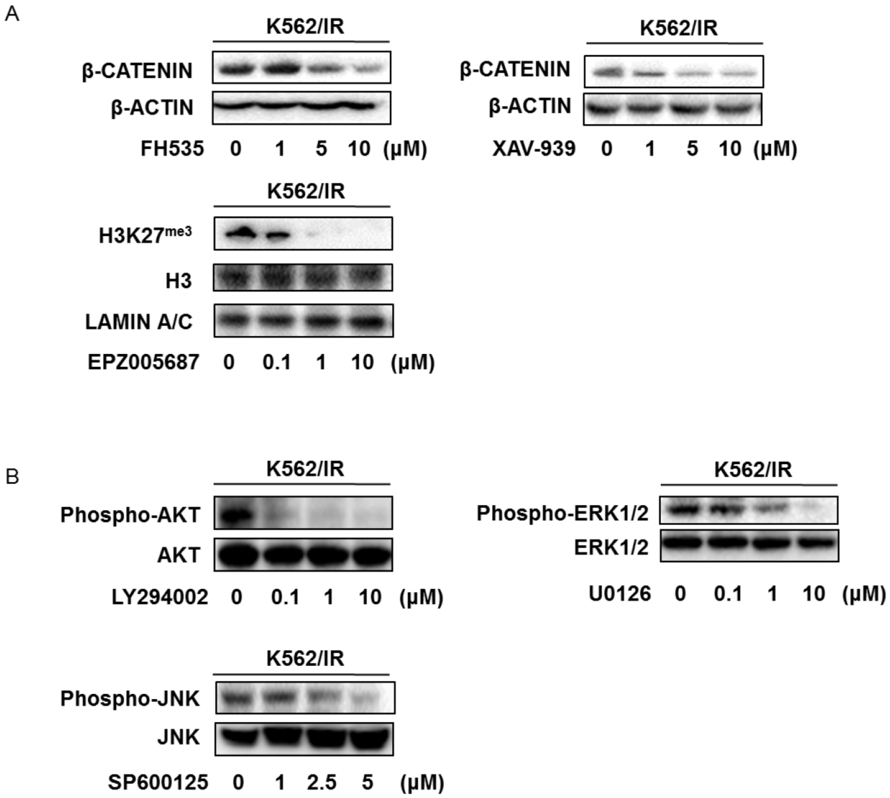

Supplementary Figure 9: Confirmed inhibitory effect of various signal inhibitors in K562/IR cells. K562/IR cells were exposed to the indicated concentrations of **(A)** FH535, XAV-939, or EPZ005687, and **(B)** LY294002, U0126, or SP600125. Whole-cell lysates were prepared and immunoblotted with antibodies against β-CATENIN, H3K27^{me3}, H3, phospho-AKT, AKT, phospho-ERK1/2, ERK1/2, phospho-JNK, JNK, β-ACTIN, or LAMIN A/C.

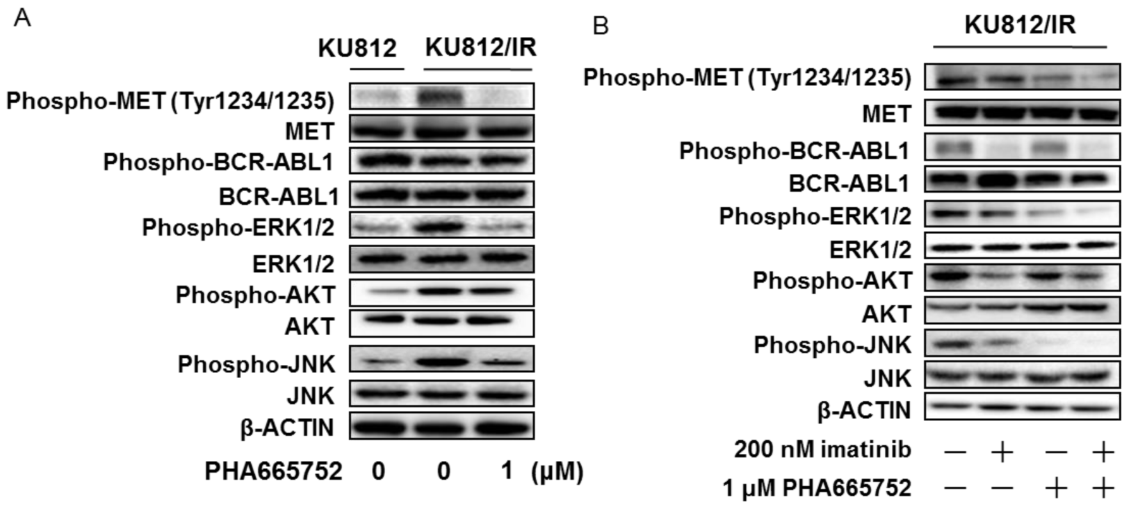

Supplementary Figure 10: MET inhibitor inhibits the ERK and JNK activation in KU812/IR cells. **(A, B)** KU812/IR cells were exposed to the indicated concentrations of imatinib or PHA665752. After incubation for 48 h, the cytoplasmic fractions were extracted and then subjected to SDS-PAGE/immunoblotting with anti-phospho-MET, anti-phospho-ABL1, anti-phospho-ERK1/2, anti-phospho-AKT, anti-phospho-JNK, anti-MET, anti-ABL1, anti-ERK1/2, anti-AKT, and anti-JNK antibodies. Anti-β-ACTIN antibody was used as internal standards.
